# Supplementary material for: A Global Screen for Assembly State Changes of the Mitotic Proteome by SEC-SWATH-MS
Source: Cell Syst. 2020 Feb 26;10(2):133–155.e6. doi: 10.1016/j.cels.2020.01.001 (PMC7042714; doi:10.1016/j.cels.2020.01.001)

Q9NPA0 | EMC7_HUMAN | EMC7 C11orf3 C15orf24 HT022 UNQ905/PRO1926
Monomer MW [kDa]: 26.471 Monomer expected elution fraction: 52

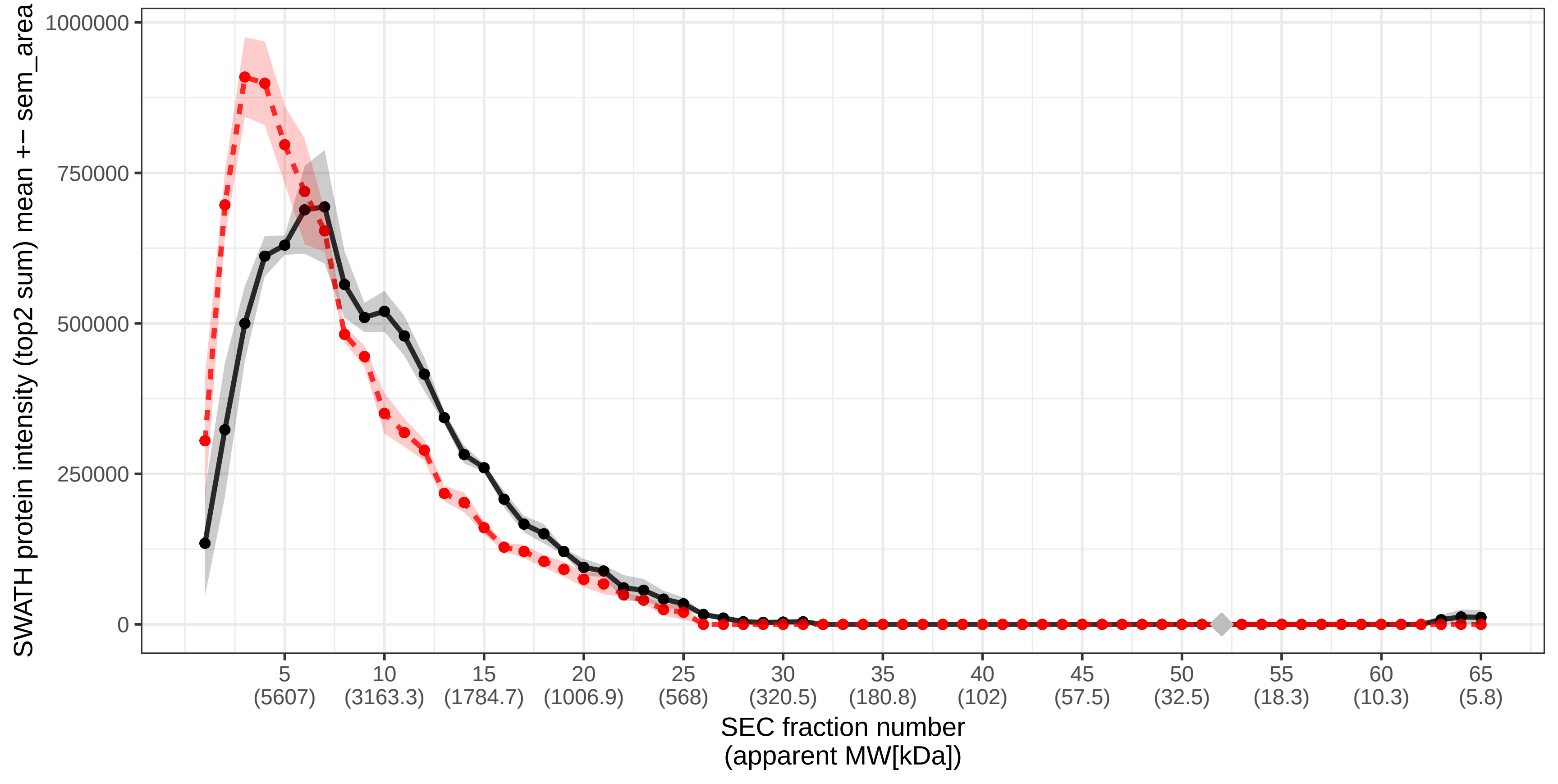

Supplement: Data S1. SEC-SWATH-MS Protein Chromatograms, Related to Figure 1 [file mmc6.zip › SECchrom_Q9NPA0_EMC7_HUMAN_EMC7_C11orf3_C15orf24_HT022_UNQ9.pdf]
